# Spatial Distribution of Medically Important Scorpions in North West of Iran

**Published:** 2017-09-08

**Authors:** Mulood Mohammadi Bavani, Javad Rafinejad, Ahmad Ali Hanafi-Bojd, Mohammad Ali Oshaghi, Shahrokh Navidpour, Farrokh Dabiri, Mehdi Badakhshan, Esmaeil Ghorbani, Masoomeh Bagheri

**Affiliations:** 1 Department of Medical Entomology and Vector Control, School of Public Health, Tehran University of Medical Sciences, Tehran, Iran; 2 Razi Reference Laboratory of Scorpion Research, Razi Vaccine and Serum Research Institute, Agricultural Research Education and Extension Organization, Karaj, Iran; 3 Department of Medical Entomology and Vector Control, School of Public Health, Urmia University of Medical Sciences, Urmia, Iran; 4 Disease Control Unit, Sareyn Health Center, Ardabil University of Medical Sciences, Sareyn, Iran

**Keywords:** Scorpions, Distribution, Morphology, GIS, Iran

## Abstract

**Background:**

Scorpions are one of the most important medical arthropods in Iran. This study aimed to determine the fauna, spatial distribution and some morphological characteristics of these venomous arthropods in the study area.

**Methods:**

Scorpions were collected using Ultra Violet light, rock rolling and digging methods in West Azerbaijan, East Azerbaijan, and Ardabil Provinces during 2015–2016. The specimens were preserved in 75% ethyl alcohol and transferred to the laboratory for species identification and morphological studies.

**Results:**

Distribution maps were produced using ArcGIS 10.3. Totally, 368 specimens from two families of Buthidae (97.1%) and Scorpionidae (2.99%) were collected and identified as *Mesobuthus eupeus* (80.16%), *Androctonus crassicauda* (10.60%), *M. caucasicus* (4.89%), *Hottentotta saulcyi* (1.35%) and *Scorpio maurus* (2.99%).

**Conclusion:**

The presence of medically important species, including the deadly black one in northwestern Iran requires health educational and control programs for reduction of these public health problems.

## Introduction

Scorpions are living fossils, and about 2000 species have been identified in the world systematically classified in different families (1). Among them, Buthidae, with a universal distribution, is the greatest family consisting of 81 genera and 570 species (2). Except for *Hemiscorpius lepturus*, all dangerous scorpion species belong to this family (3). Scorpion stings are a major public health problem in subtropical and tropical countries (4) and some species can be lethal for human. Annually, nearly 1230000 cases of scorpion sting with a fatality rate of 27% are reported in different parts of the world (5). The most medically important species belong to *Hottentotta*, *Buthus*, *Tityus*, *Leiurus*, *Androctonus*, *Parabuthus*, *Centruroides* and *Mesobuthus* genera (6).

Scorpions’ venom is a complex mixture containing peptides, enzymes, mucoproteins, free amino acids, nucleotides, lipids, amines, heterocyclic components, non-organic salts and low molecular-weight organic molecules. Peptides that affect ion channels including sodium, potassium, calcium and cholera channel are called neurotoxin, and because of their effect on ion channels endanger human health and sometimes results in death (7). Two Iranian dangerous scorpions’ families i.e. *Buthidae* and *Hemiscorpiidae* have different effects on human; such that *Buthidae* species have mostly neurotoxic effect, but *Hemiscorpiidae* such as *Hemiscorpius lepturus* (local name: Gadim), causes cell death and hemolytic effect cause fatality rate in the country (8, 10).

Because of the remarkable diversity in zoogeographic zones of Iran, there is a rich arthropod’s fauna that includes scorpions. Several dangerous species inhabit some areas across different parts of Iran, and some of them are lethal for human (11). Scorpion stings cases have been reported as exceeding 42500 annually in Iran, with a fatality rate of 44.7 per 100000. These figures put the country in the second place after Mexico concerning case fatality rate (11, 12). These numbers are not definitive and the actual numbers of scorpion sting and fatality rate are greater than the annual recorded figures.

Olivier studied the scorpion fauna in Iran and described the black scorpion (*Androctonus crassicauda*) in Kashan City (13). Since then, several studies have reported 4 families, 19 genera and 53 species of scorpions in Iran. Buthidae, consisting of 15 genera and 45 species, is the largest family (14, 31).

*Mesobuthus eupeus*, *A. crassicauda*, *H. lepturus*, *Odontobuthus doriae*, *H. saulcyi*, *Hottentotta schach*, *Compsobuthus matthiesseni* and *M. caucasicus* are the most medically important species, while *A. crassicauda* and *H. lepturus* are the most dangerous and deadly species to human in Iran (11, 32, 34).

Due to the larger extent and the importance of scorpions in the southern tropical areas of the country, most studies have been performed in this area. Therefore, faunistic, morphological, spatial distribution and ecological studies on these toxic arthropods have been neglected in other parts of the country.

There is a few information concerning species composition, geographical distribution and the morphology of scorpions in the northwestern corner of Iran. Report of an annual number of 1186 scorpions sting cases in this area (1) encouraged us to design and conduct this study. Therefore, this investigation conducted to clarify the current fauna, abundance, geographical distribution and some morphological features of scorpions to provide identification key and its control guideline in this region of the country.

## Materials and Methods

### Study area

**Fig. 1.**
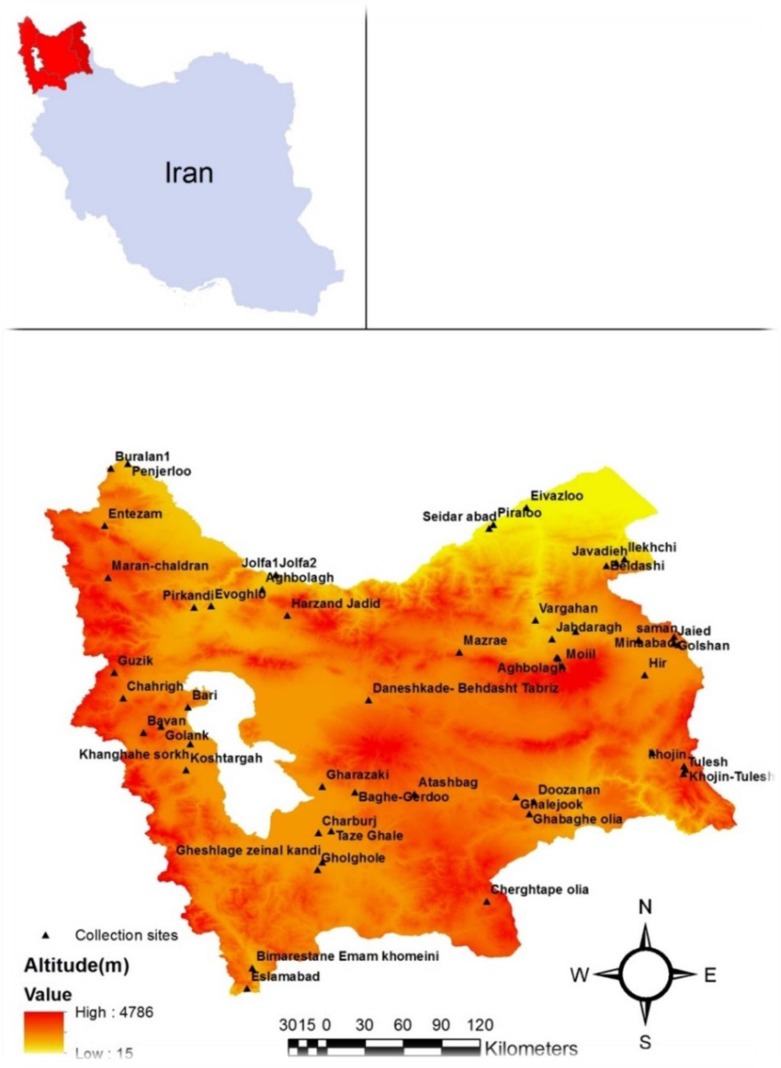
Study area in north west of Iran

This study was conducted in West Azerbaijan, East Azerbaijan and Ardabil Provinces, all in North West part of Iran. These provinces cover an area of about 1007031 km^2^, between 35°58′–39°46′N latitudes and 44°03′–48°55′E longitudes. They share borders with Armenia and Republic of Azerbaijan in the north, Kurdish inhabited area in Turkey and Kurdistan regional government in the west, Gilan Province in the east, and Zanjan and Kurdistan Provinces in the south. These provinces include 43 counties with a total population of 8053684 inhabited this area in various weather and environmental conditions. The region has both plains and mountains (Fig. 1).

### Specimen and data collection

Fifty-four locations were selected and the scorpion collection and sampling procedure was carried out from Jun to Aug 2015–2016 during day and night periods using the standard methods, i.e. rock-turning (35) (searching under the stones and rock etc.) and UV lights (36). In addition, to capture burrowing scorpions, pouring water in holes, digging and rubber band technique were used in various scorpions’ habitats.

Geographical location, latitude, longitude, altitude (using GPS), environmental temperature and relative humidity (using digital thermometer and hygrometer) were recorded during sampling. All samples were preserved in 75% ethyl alcohol and kept in the different labeled plastic boxes according to their localities, and then transferred to the School of Public Health, Tehran University of Medical Sciences, and Tehran, Iran.

Morphological identification and analyses such as dentition of the pedipalp chela on movable finger, accessory granules under the terminal denticle on the pedipalp chela movable finger (AGMF), rows of denticles on the pedipalp chela movable finger (RDMF), pectinal teeth (PT) were done when observed under stereomicroscope using standard Iranian scorpion identification keys (12,14). ArcGIS 10.3 was used to produce the distribution maps of the collected species.

## Results

**Fig. 4.**
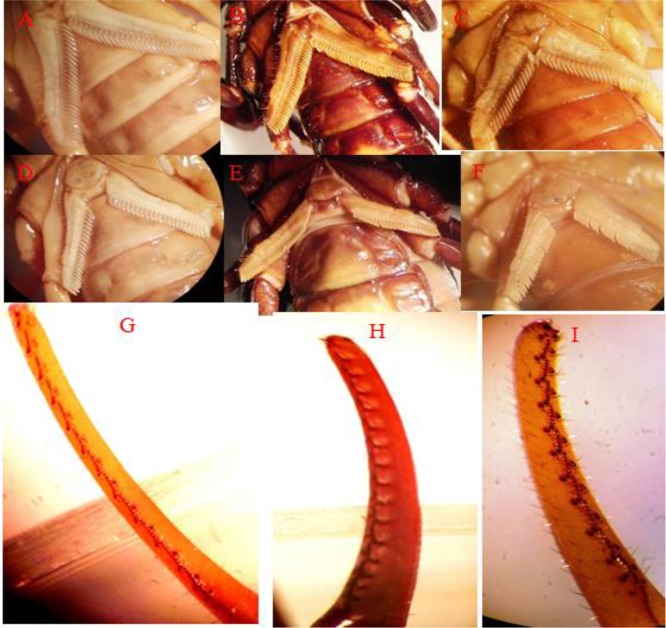
Morphological characteristic of medically important collected scorpions from north west of Iran. (A–C) Pectinal teeth of adult male, (A) *Mesobuthus caucasicus*, (B) *Androctonus crassicauda*, (C) *Mesobuthus eupeus*; (D–F) Pectinal teeth of adult female. (D) *Mesobuthus caucasicus*, (H) *Androctonus crassicauda*, (F) *Mesobuthus eupeus*. (G–I) Rows of denticles on the pedipalp chela movable finger. (G) *Mesobuthus caucasicus*, (H) *Androctonus crassicauda*, (I) *Mesobuthus eupeus*

In total, 368 scorpions were collected and identified. They belonged to two families, five genera and five species: *M. eupeus* (80.16%), *A. crassicauda* (10.60%), *M.* (*Olivierus*) *caucasicus* (4.89%), *S. maurus* (2.99%) and *H.* (*Buthotus*) *saulcyi* (1.35%) (Fig. 4). Except for *S. maurus* (Scorpionidae), other species belonged to the Buthidae family. Table 1 shows the species composition and sex ratio of the scorpions collected from different counties of the study area.

**Table 1.** Species composition and spatial distribution of scorpions collected in northwest of Iran

	**Species**										**Total(N)**	

	***M. eupeus***		***A. crassicauda***		***M. caucasicus***		***S. maurus***		***H. saulcyi***			

**Location**	**Male(N)**	**Female(N)**	**Male(N)**	**Female(N)**	**Male(N)**	**Female(N)**	**Male(N)**	**Female(N)**	**Male(N)**	**Female(N)**	**Male(N)**	**Female(N)**
**Parasad**	18	20	-	-	-	-	-	-	-	-	18	20
**Germi**	4	6	-	-	-	-	-	-	-	-	4	6
**Namin**	27	16	-	-	-	-	-	-	-	-	27	16
**Ardabil**	4	8	-	-	-	-	-	-	-	-	4	8
**Meshkinshahr**	17	21	-	-	-	-	-	-	-	-	17	21
**Givi**	-	-	-	-	-	-	1	-	-	-	1	-
**Khakhal**	3	7	-	-	-	-	-	-	-	-	3	7
**Khodaafarin**	10	14	-	-	-	-	-	-	-	-	10	14
**Ahar**	3	3	-	-	-	-	-	-	-	-	3	3
**Mianeh**	5	8	-	-	-	-	-	-	-	-	5	8
**Hashtrood**	1	-	-	-	-	-	-	-	-	-	1	-
**Bonab**	-	-	2	2	1	-	-	-	-	-	3	2
**Marand**	3	-	-	-	1		-	-	-	-	3	-
**Jolfa**	9	6	2	-	2	-	-	-	-	-	13	6
**Malakan**	-	-	-	-	1	-	-	-	-	-	1	-
**Tabriz**	-	-	-	-	3	1	-	-	-	-	3	1
**Maraghe**	-	-	1	-	-	-	-	-	-	-	1	
**Khoi**	2	1	4	2	-	-	-	-	-	-	6	3
**Makoo**	2	2	1	1	-	-	-	-	-	-	4	3
**Salmas**	3	5	-	-	3	1	-	-	-	-	6	6
**Urmia**	27	36	17	6	3	1	-	-	-	-	47	43
**Mahabad**	-	1	-	-	-	-	-	1	-	-	-	2
**Miandoab**	-	-	-	-	1	-	1	9			2	9
**Sardasht**	-	-	1	-	-	-	-	-	-	5	3	3
**Tekab**	2	1	-	-	-	-	-	-	-	-	2	1

**Total**	140	155	28	11	15	3	1	10	-	5	186	182

### Morphological characteristics of scorpions

**Fig. 2.**
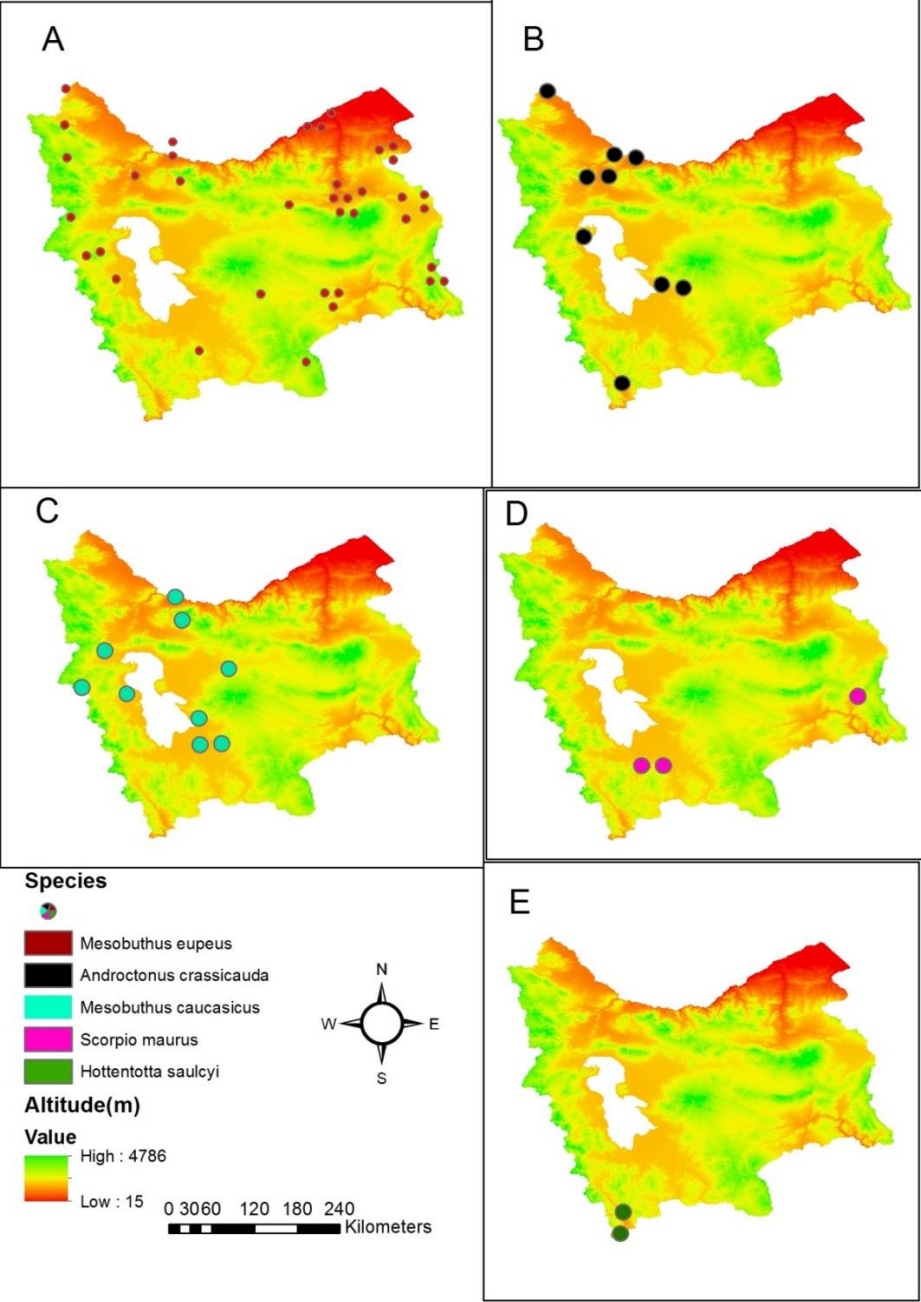
Spatial distribution of collected scorpions from northwestern Iran. (A) *Mesobuthus eupeus*, (B) *Androctonus crassicauda*, (C) *Mesobuthus Caucasicus*, (D) *Scorpio maurus*, (E) *Hottentotta saulcyi*.

**Fig. 3.**
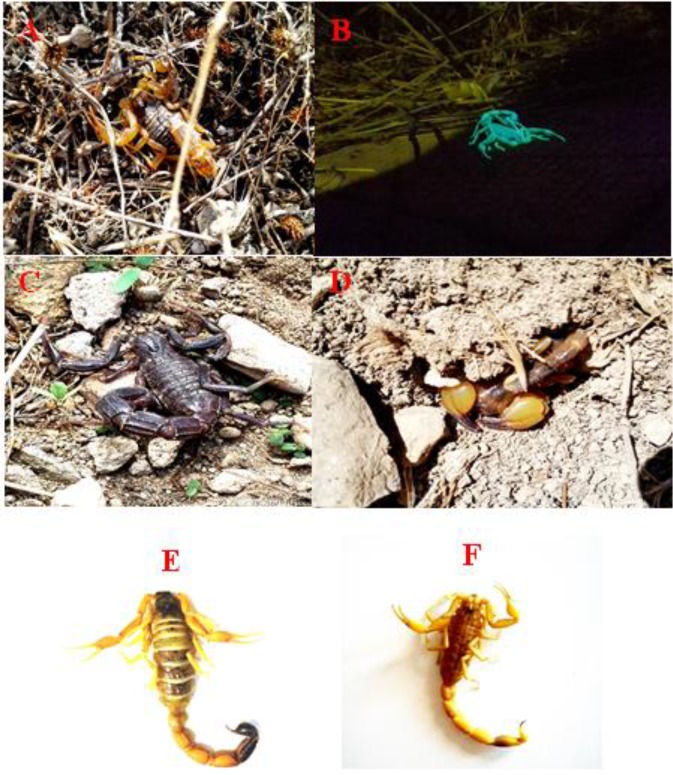
Collected scorpions’ species form north west of Iran: (A) *Mesobuthus euepeus*, (B) *Hottentotta saulcyi* under UV light, (C) *Androctonus crassicauda*, (D) *Scorpio maurus*, (E) *Hottentotta saulcyi*, (F) *Mesobuthus caucasicus*

***M. eupeus*** (Fig. 3A). In total, 295 specimen (80.16% of the collected samples) of this species were captured in the 34 localities of all three provinces (West Azerbaijan, East Azerbaijan and Ardabil Provinces), (Fig. 2A).

This non-digger species belongs to the family of Buthidae may be regarded as a complex species. The variation in color pattern was observed from north to the south so that the northern specimens near the Aras River had darker spots and stripes on their cara-pace and mesosomal segments than others.

This species was found in all the three provinces with a wide distribution from Parsabad in the North, to Tekab in the South, and from Urmia in the West to Namin in the East (Fig. 2A).

This species was found in different localities and habitats with different climates in urban and rural area including under stones and bark, on the farm and plow, near houses, in cemetery, dilapidated homes and grasslands. This species was collected from plains and mountainous area with an altitude range between 188 to 2180m.

**Fig. 5.**
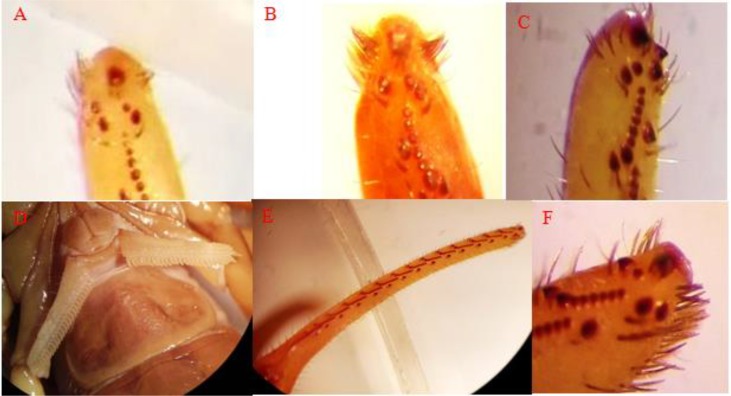
**(A–C)** Number of accessory granules under the terminal denticle on the pedipalp chela movable finger. (A) *Mesobuthus caucasicus*, (B) *Androctonus crassicauda*, (C) *Mesobuthus eupeus*, (D–F) *Hottentotta saulcyi* adult female. (D) Pectinal teeth, (E) Rows of denticles on the pedipalp chela movable finger, (C) Number of accessory granules under the terminal denticle on the pedipalp chela movable finger

The sex ratio for this species was 1:1.10 in favor of females. Pectineal teeth (Fig. 4C, F) were between 21/22–30/32 for males and 16/17–23/25 for females; the number of accessory granules under the terminal denticle on the pedipalp chela movable finger (Fig. 5C) was 4 for both sexes but seldom had they 0, 2, 3 and 5 granules. Rows of denticles on the pedipalp chela movable finger (Fig. 4I) were 12–14.

***A. crassicauda*** (Fig. 3C). Totally, 39 specimen from 9 localities of two provinces (West Azerbaijan and East Azerbaijan) (Fig. 2B) were collected, comprising 10.6% of all captured scorpions.

This species inhabited indoors of buildings and hospitals, under stones, inside soil, sand and cemetery stones in plain and mountain areas at an altitude ranging from 690 to 1473m. This is the second most common and deadly scorpion species collected in some counties, but was absent in the Ardabil Province.

Pectinal teeth (Fig. 4B, E) were 31/32–34/35 for males and 23/24–26/27 for females. Rows of denticles on the pedipalp chela movable finger (Fig. 4H) were 15–16 for females and 13–16 for males. The sex ratio was 1:0.39 in favor of males. The numbers of granules under the end granule (Fig. 5B) were 3, but sometimes in a few specimens only one was observed, like those of *M. eupeus*, this is uncommon.

***M. caucasicus*** (Fig. 3F). In total, 18 specimens (4.89% of all collected samples) of this species were captured in 9 locations of two provinces (West Azerbaijan and East Azerbaijan) (Fig. 2C) in mountainous areas with a height of 690–2180m.

This species always seek shelter under stones and inside the wooden roofs of rural houses. Pectinal teeth (Fig. 4A and 4D) and number of rows of denticles on the pedipalp chela movable finger (Fig. 4G) were 26/28–31/29 and 22/23–23/24 and 11–14 and 12–13 for males and females, respectively. The sex ratio was 1:0.2 in favor of males. The numbers of granules under the end granule on movable finger (Fig. 5A) were 4 in all specimens.

***S. maurus*** (Fig. 3D). This digger species belongs to the family of Scorpionidae and was found only in Givi (Ardabil Province), Mahabad and Miandoab (West Azerbaijan Province) (Fig. 2D) Counties at the heights of 1286, 1404 and 1431m, but this species was not collected from East Azerbaijan Province. The sex ratio was 1:10 in favor of females.

***H. saulcyi*** (Fig. 3E). This species has limited distribution in the region in which it was collected. We collected it only from two sites of Sardasht County, West Azerbaijan Province, at the heights of 1286, 1404 and 1431m (Fig. 2E). This species was not caught in the Ardabil and East Azerbaijan Provinces. Only the females of this taxon were captured.

Pectinal teeth (Fig. 5D), the number of dens row on movable finger (Fig. 5E) and granules under the end (Fig. 5F) were 24/25–26/27 and 14–15, and 4, respectively.

## Discussion

In this study, 368 specimens were collected comprising five species that include *M. eupeus*, *A. crassicauda*, *O. caucasicus*, *S. maurus* and *H. saulcyi*. Buthidae is the largest family of scorpions in the world, as well as in Iran, and this species was documented as the largest family of the collected scorpions in this study. Buthidae was the largest family (97.1% of total), and *M. eupeus* and *A. crassicauda* are the most medically abundant species in the northwestern quarter of the country. Similar to our results, some recent investigation have reported Buthidae as the largest family comprising 88.5% of all the species, and *A. crassicauda* and *M. eupeus* as the most abundant, medically important species (32, 37).

The genus *Hemiscorpius* has the first fatality rate in Iran, followed by the genus *Androctonus*. Although we could not find any species of the first genus, *A. crassicauda* was collected from East Azerbaijan and West Azerbaijan. Two cases of death probably from this black deadly scorpion were reported from the study area (38). Hence, health staffs should be familiar and well educated to know scorpions’ morphology and ecology, educate the community on how to prevent and treat scorpion’s sting.

In our study, the family Buthidae comprises 97.1% of all collected specimen, among which *M*. *eupeus* was the most prevalent and included 80.16% of all specimen, and this species is regarded as the most medically important scorpion species in the region. This is because of the wider geographical distribution of *M. eupeus*, which accounts for the majority of scorpion envenomation in this part of Iran.

This species is considered as most common scorpion in all parts of Iran, including the Northwestern regions, and inhabits areas under stones, tree barks, construction debris, in the roof of old buildings and inside beetle nests (as observed in this study). These species have about 14 members through its geographical distribution area, and this species can be a complex species (39, 40).

There are six members, however, only one member of the species (*Mesobuthus eupeus eupeus*) has been reported in our study area (39). These studies were limited to one or two counties in the north west of Iran and had not covered the entire area (39).

In comparison, morphological diversity of this species was recognized in our study, at least in coloration pattern, so that the color of northern species near the Aras River was darker (in Carapace and Mesosoma) than those found in the central and southern parts, for examples: Urmia and Tekab. To prove these differences, molecular experiments should be done.

The second most prevalent species, *A. crassicauda*, is the most dangerous and deadly scorpion in Iran, especially the northwestern parts, such that (this study) its sting cause local pain and autonomic stimulation and fatal cardiovascular problems, particularly in children, and needs special attention because its domestic activity is moving towards human shelters (32).

Other collected species included *M. caucasicus*, *H. saulcyi* and *S. maurus*, among them, the first and second are classified as belonging to Iranian medically important species. *Mesobuthus caucasicus* is regarded as the third medically important species due to its wide dispersion and low density in the region. *H. saulcyi* is considered as the fourth medically important species in the region due to its limited distribution.

Studies were conducted in Zanjan Province that shares long borders with our study area and have collected *A. crassicauda*, *H. saulcyi*, *Hottentotta zagrosensis*, *O. doriae*, *M. eupeus* and *S. maurus.* Among them, four species including *M. eupeus*, *A. crassicauda*, *H. saulcyi* and *S. maurus* are common between this province and our study region (29).

In addition, in Turkey which have long borders with Western Azerbaijan Province disclosed the presence of *L. quinquestriatus*, *M. gibbosus* and *M. eupeus* of the family Buthidae, among which *M. eupeus* and *A. crassicauda* are common in our study (41, 44).

The polyvalent antivenom in Iran is produced by the Razi Vaccine, Serum, and Research Institute against the 6 medically important scorpion species: *M. eupeus*, *A. crassicauda*, *O.* and *H. saulcyi*.

Granules under the terminal denticle on the pedipalp chela movable finger were 4 (Fig. 5C) for all specimens, however, many variations were observed in our study. Rows of denticles on the pedipalp chela movable finger (Fig. 4I) were 11, but a range of 12–14 was observed in our study, and these variations show that probably there are more than one types of this species in northwestern provinces of Iran. This requires the necessity of molecular experiments to clarify which kind of this species is present in these areas.

Only females of *H. saulcyi* were captured with pectinal teeth of 24/25–26/27, but the female specimen from Turkey and Iraq had 26–28 pectinal teeth and Iranian females’ specimens had 27–28. The number of granules on the tip of the movable finger was 3–5 (in Turkey and Iraq specimens), in Iran was 4 and sometimes was absent (45, 46). However, in our study, all collected specimens had 4 granules and 14–15 rows of denticles on Chela movable finger.

The number of pectinal teeth was investigated to distinguish between the genders of scorpions (46). The number of pectinal teeth (PT) of *A. crassicauda* was 30 and 24 in males and females of this scorpion, respectively (47). These findings were in contrast with our study where PT varied between 31/32–34/35 for males and 23/24–26/27 for females.

A study conducted in Zarrindasht County, Fars Province of Iran, showed sex ratios (male to female) of *S. maurus*, *M. caucasicus* and *M. eupeus* to be 1:5.73, 1:0.31 and 1:0.55, respectively. Similarly, our study recorded the sex ratio of *M. eupeus* and *M. caucasicus* in favor of males. Males of captured scorpions were more prevalent than females, but for *S. maurus* this ratio was in favor of females (1:10) than males (48).

## Conclusion

There are four medically important scorpion species including *M. eupeus*, *A. crassicauda*, *M. caucasicus* and *H. saulcyi* in northwestern Iran, among them, *A. crassicauda* is the most deadly scorpion in the study area. Hence, residents should be careful of its sting, especially in children.

Another medically important, but not deadly, species is *M. eupeus* which inhabited all studied provinces. Species composition and spatial distribution of scorpions, clarified in the northwestern provinces of Iran will be useful to assign the appropriate share of monovalent antivenom to the area and will reduce the public health problem and mortality due to scorpion sting because of quick access to treatment.
